# Supplementary material for: Long-read proteogenomic atlas of human neuronal differentiation reveals isoform diversity informing neurodevelopmental risk mechanisms
Source: bioRxiv. 2025 Dec 19:2025.12.16.693263. Preprint. [Version 1] doi: 10.64898/2025.12.16.693263 (PMC12724457; doi:10.64898/2025.12.16.693263)
Supplement: Supplement 8 [file NIHPP2025.12.16.693263v1-supplement-8.pdf]

bioRxiv preprint doi: https://doi.org/10.64898/2025.12.16.693263; this version posted December 19, 2025. The copyright holder for this preprint (which was not certified by peer review) is the author/funder, who has granted bioRxiv a license to display the preprint in perpetuity. It is made available under aCC-BY 4.0 International license.

Supplemental Figures

Fig. S1

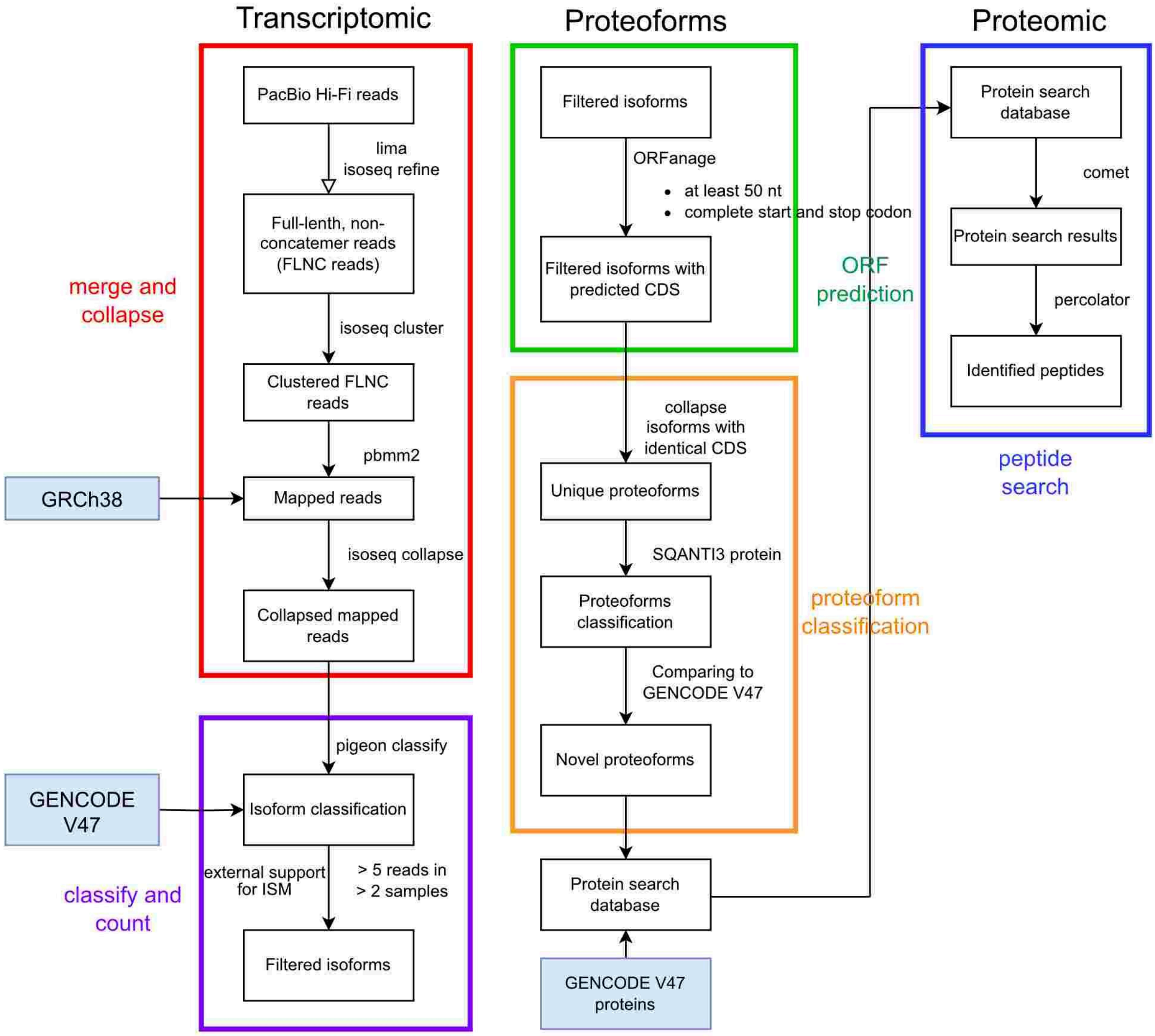

Figure S1. An overview of the proteogenomic bioinformatics pipeline. This flowchart details the processing of raw long-read transcriptomic data (red box) and its classification against GENCODE V47 to define a high-confidence set of filtered isoforms (purple box). This transcriptome is then used to predict open reading frames (ORFs) (green box) and classify novel proteoforms (orange box). These novel proteoforms are combined with reference proteins to create a custom database used for the proteomic peptide search (blue box) to validate translation. Further details are provided in Methods.

bioRxiv preprint doi: https://doi.org/10.64898/2025.12.16.693263; this version posted December 19, 2025. The copyright holder for this preprint (which was not certified by peer review) is the author/funder, who has granted bioRxiv a license to display the preprint in perpetuity. It is made available under aCC-BY 4.0 International license.

Fig. S2

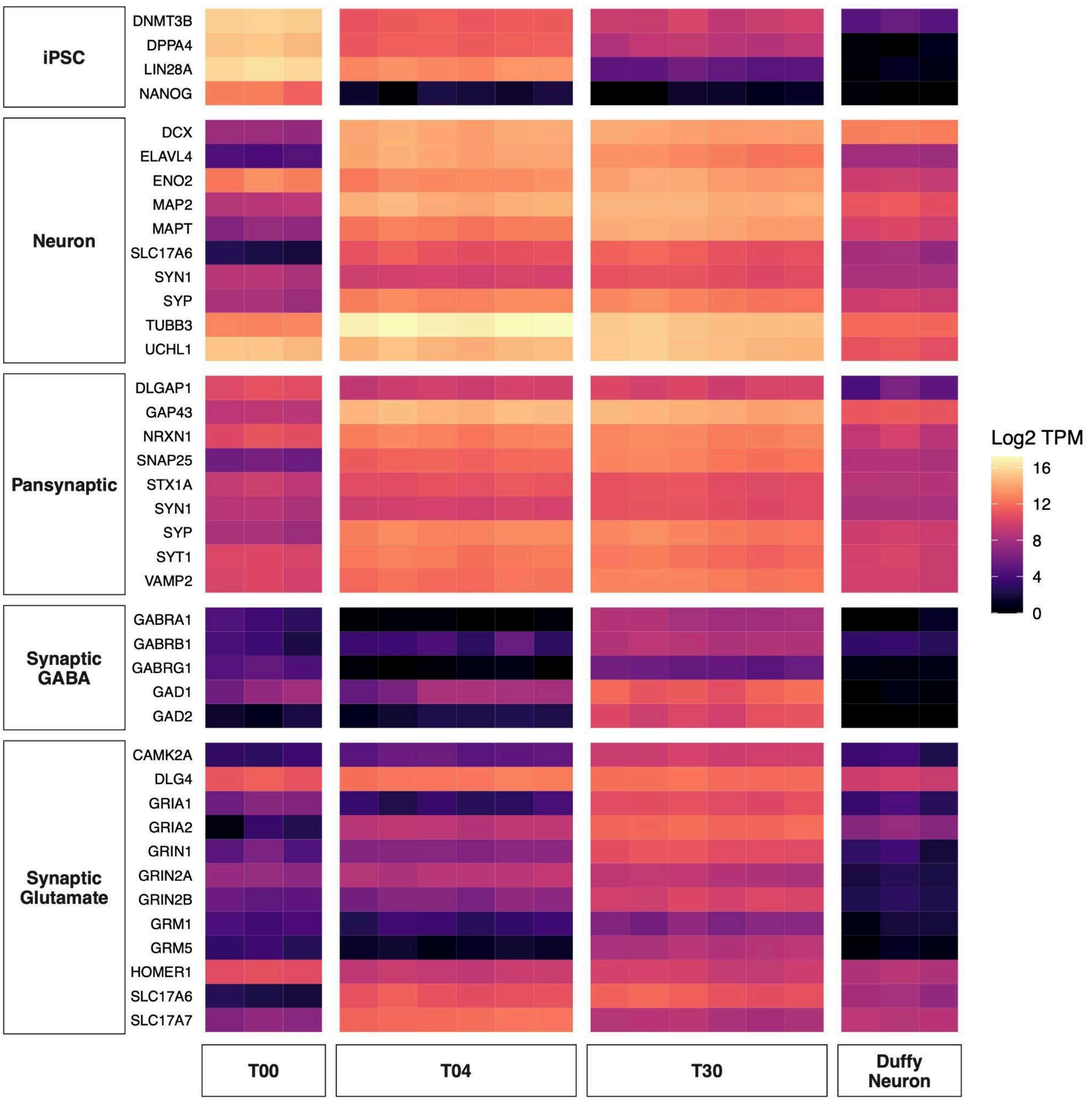

Figure S2. Expression of canonical cell-type markers confirms cellular identity across the differentiation time course. The heatmap shows gene expression (Log2 TPM) from this study's short-read RNAseq data at t00 (iPSC), t04 (intermediate), and t30 (neuron) time points. The final column displays expression from an external NGN2-based iPSC-derived neuron dataset for comparison[25].

bioRxiv preprint doi: https://doi.org/10.64898/2025.12.16.693263; this version posted December 19, 2025. The copyright holder for this preprint (which was not certified by peer review) is the author/funder, who has granted bioRxiv a license to display the preprint in perpetuity. It is made available under aCC-BY 4.0 International license.

Fig. S3

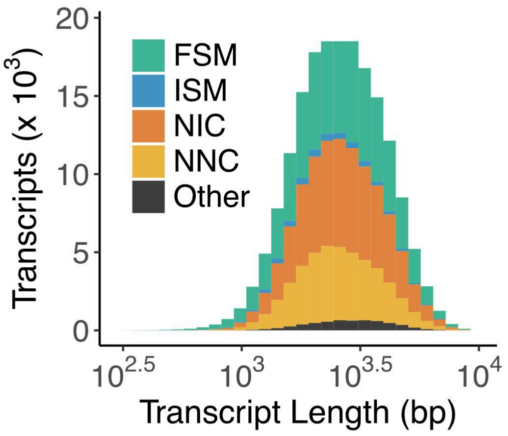

Figure S3. Distribution of isoform lengths from long-read RNAseq. Related to Fig. 1. This plot shows that isoforms of different structural categories have a similar distribution of transcript length that centres around 2600 bp.

Fig. S4

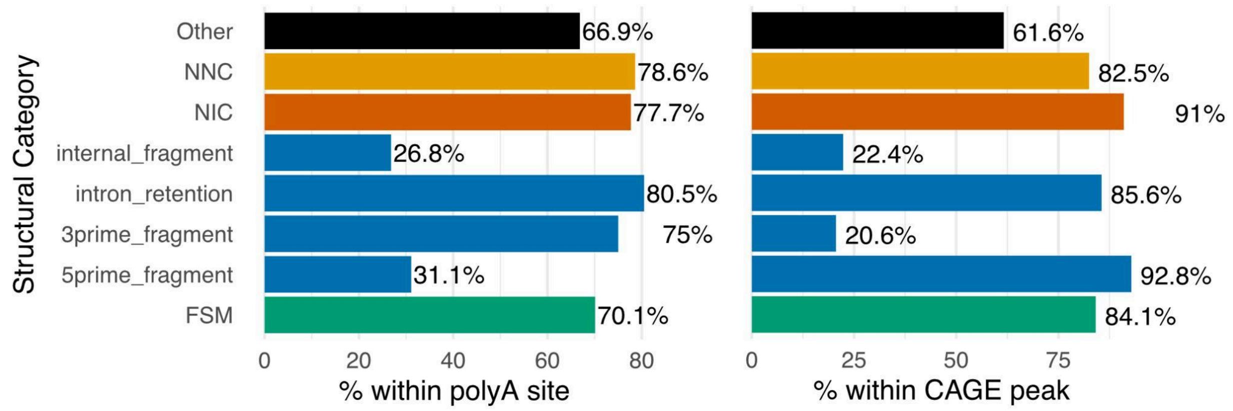

Figure S4. Validation of isoform 3' and 5' ends using external datasets. Related to Fig. 1. Bar plots show the percentage of isoforms whose 3' ends overlap a known polyA site (left) and whose 5' ends overlap a CAGE peak (right). Structural categories match Fig. 1, with Incomplete Splice Matches (ISM) further stratified into four subcategories: internal fragments, intron retention, 3' fragments (putative 5' truncation), and 5' fragments (putative 3' truncation). To minimize technical artifacts, only 3' fragments with valid 5' support (CAGE peaks) and 5' fragments with valid 3' support (polyA sites) were retained for the final high-confidence catalog.

bioRxiv preprint doi: https://doi.org/10.64898/2025.12.16.693263; this version posted December 19, 2025. The copyright holder for this preprint (which was not certified by peer review) is the author/funder, who has granted bioRxiv a license to display the preprint in perpetuity. It is made available under aCC-BY 4.0 International license.

Fig. S5

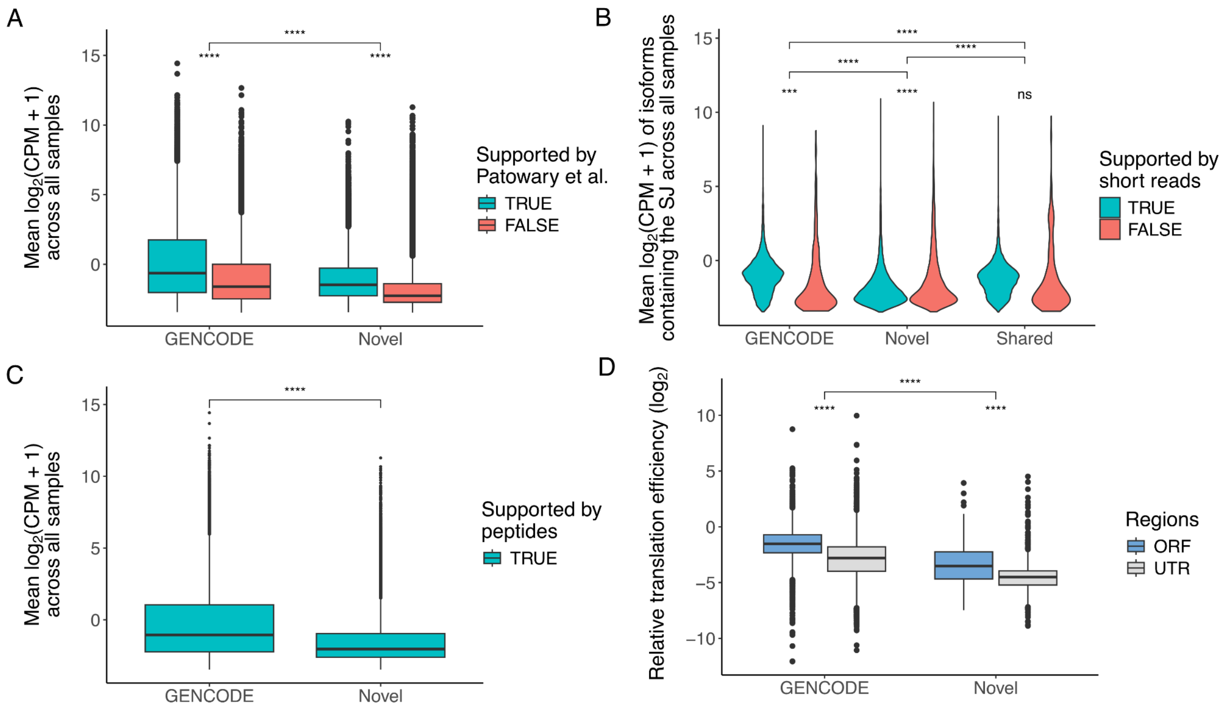

Figure S5. Transcript abundance is a major contributor to multi-omic validation of novel isoforms. (A) Mean expression (log2 CPM+1) of GENCODE and Novel isoforms, stratified by their detection in an external fetal brain long-read RNAseq dataset from Patowary et al., 2024. Isoforms replicated in Patowary et al have significantly higher expression than those unique to this study. **(B)** Mean expression of isoforms containing GENCODE, Novel, or Shared splice junctions, stratified by support from our matched short-read RNAseq. Validated junctions (teal) are associated with significantly higher isoform expression. **(C)** Mean expression of full-length isoforms supported by peptides from proteomics; where a peptide maps to multiple isoforms, expression is averaged across all isoforms containing peptide. Validated novel isoforms exhibit significantly lower expression than validated known isoforms. **(D)** Relative translational efficiency (TE), defined as the ratio of ribosome-protected fragments to input mRNA, calculated using external Ribo-seq data from human iPSC-derived neurons from Duffy et al., 2022. As a positive control, TE is significantly higher in known GENCODE coding sequences compared to 3'UTR. This pattern extends to the novel isoforms identified here, where predicted ORFs exhibit significantly greater TE compared to predicted 3'UTRs, providing independent validation for the ribosomal engagement and translation of these novel coding sequences. ***P < 0.001; ****P < 0.0001; ns = not significant.

bioRxiv preprint doi: https://doi.org/10.64898/2025.12.16.693263; this version posted December 19, 2025. The copyright holder for this preprint (which was not certified by peer review) is the author/funder, who has granted bioRxiv a license to display the preprint in perpetuity. It is made available under aCC-BY 4.0 International license.

Fig. S6

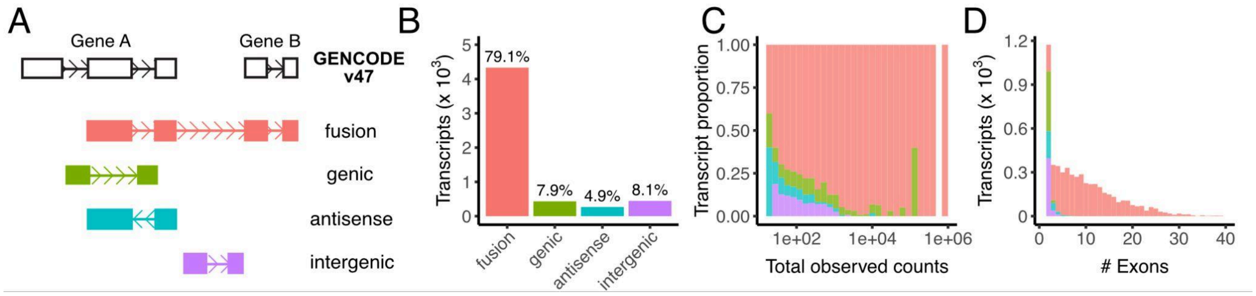

Figure S6. Classification and characterization of fusion, genic, antisense, and intergenic transcripts. (A) Schematic illustrating the classification of transcripts relative to the GENCODE V47 reference. Categories are defined as 'fusion' (spanning two distinct gene loci), 'genic' (overlapping a known gene), 'antisense' (on the opposite strand of a known gene), and 'intergenic' (in a previously unannotated region). (B-D) Characterization of these transcript categories. These structural categories are used to illustrate the total count and proportion of all high-confidence transcripts (B), as well as the distributions of total read counts (C) and exon counts (D) per transcript.

bioRxiv preprint doi: https://doi.org/10.64898/2025.12.16.693263; this version posted December 19, 2025. The copyright holder for this preprint (which was not certified by peer review) is the author/funder, who has granted bioRxiv a license to display the preprint in perpetuity. It is made available under aCC-BY 4.0 International license.

Fig. S7

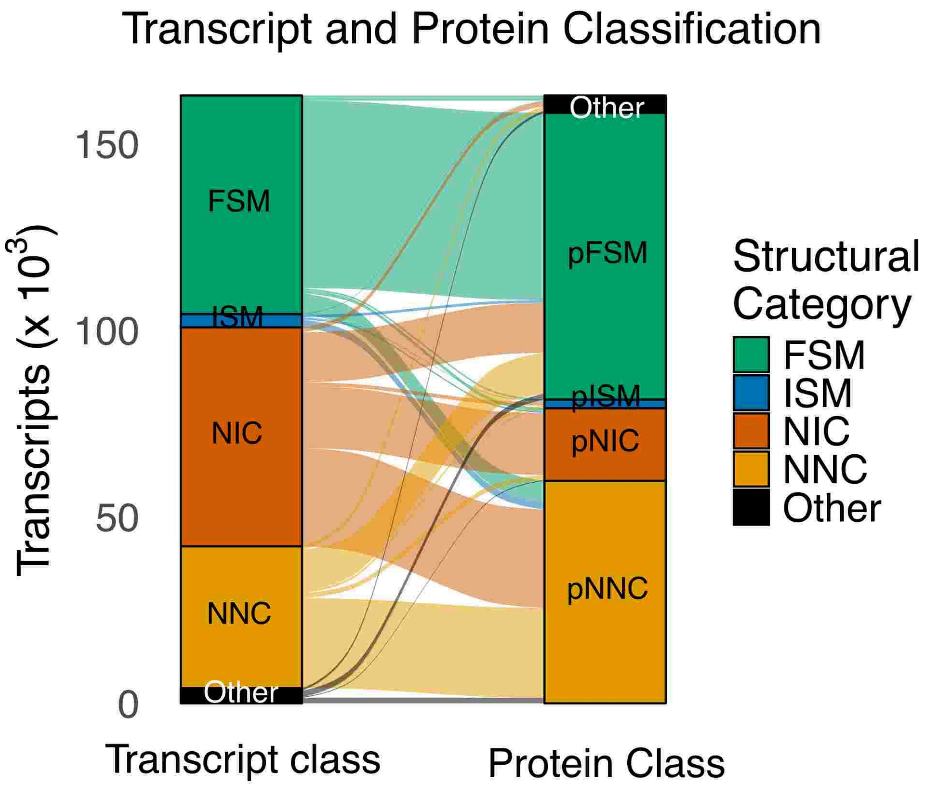

Figure S7. Relationship between transcript and protein structural classifications. Sankey diagram illustrating the mapping between the structural classification of mRNA isoforms and their corresponding predicted protein isoforms, using the SQANTI protein framework.

bioRxiv preprint doi: https://doi.org/10.64898/2025.12.16.693263; this version posted December 19, 2025. The copyright holder for this preprint (which was not certified by peer review) is the author/funder, who has granted bioRxiv a license to display the preprint in perpetuity. It is made available under aCC-BY 4.0 International license.

Fig. S8

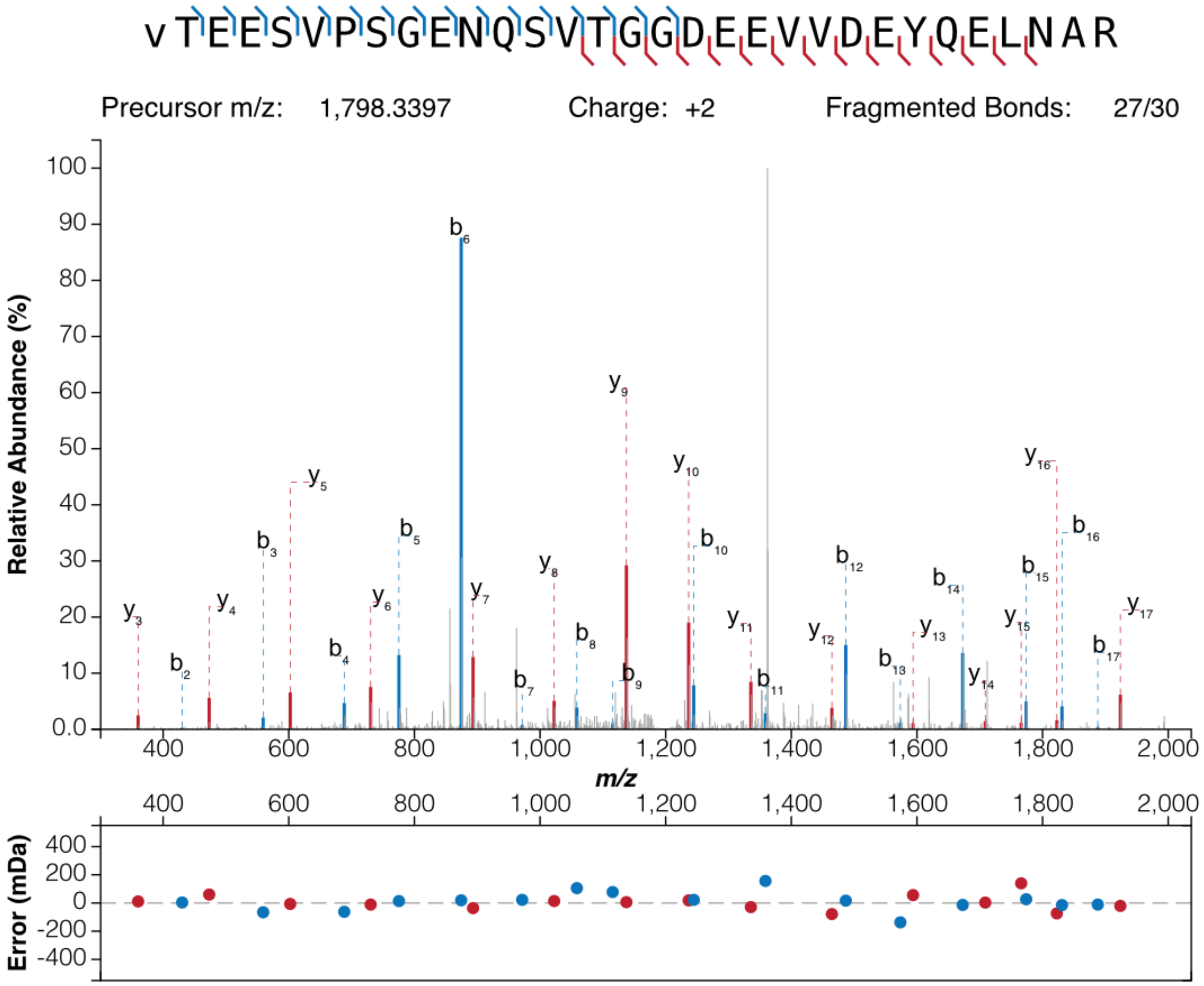

Figure S8. Peptide confirmation of the translation of a novel exon in EXOC1. Related to Fig. 2. Mass spectrum of peptide VTEESVPSGENQSVTGGDEEVVDEYQELNAR, which confirms the translation of the detected *EXOC1* microexon. Matched b and y ions are highlighted. m/z, mass/charge ratio.

bioRxiv preprint doi: https://doi.org/10.64898/2025.12.16.693263; this version posted December 19, 2025. The copyright holder for this preprint (which was not certified by peer review) is the author/funder, who has granted bioRxiv a license to display the preprint in perpetuity. It is made available under aCC-BY 4.0 International license.

Fig. S9

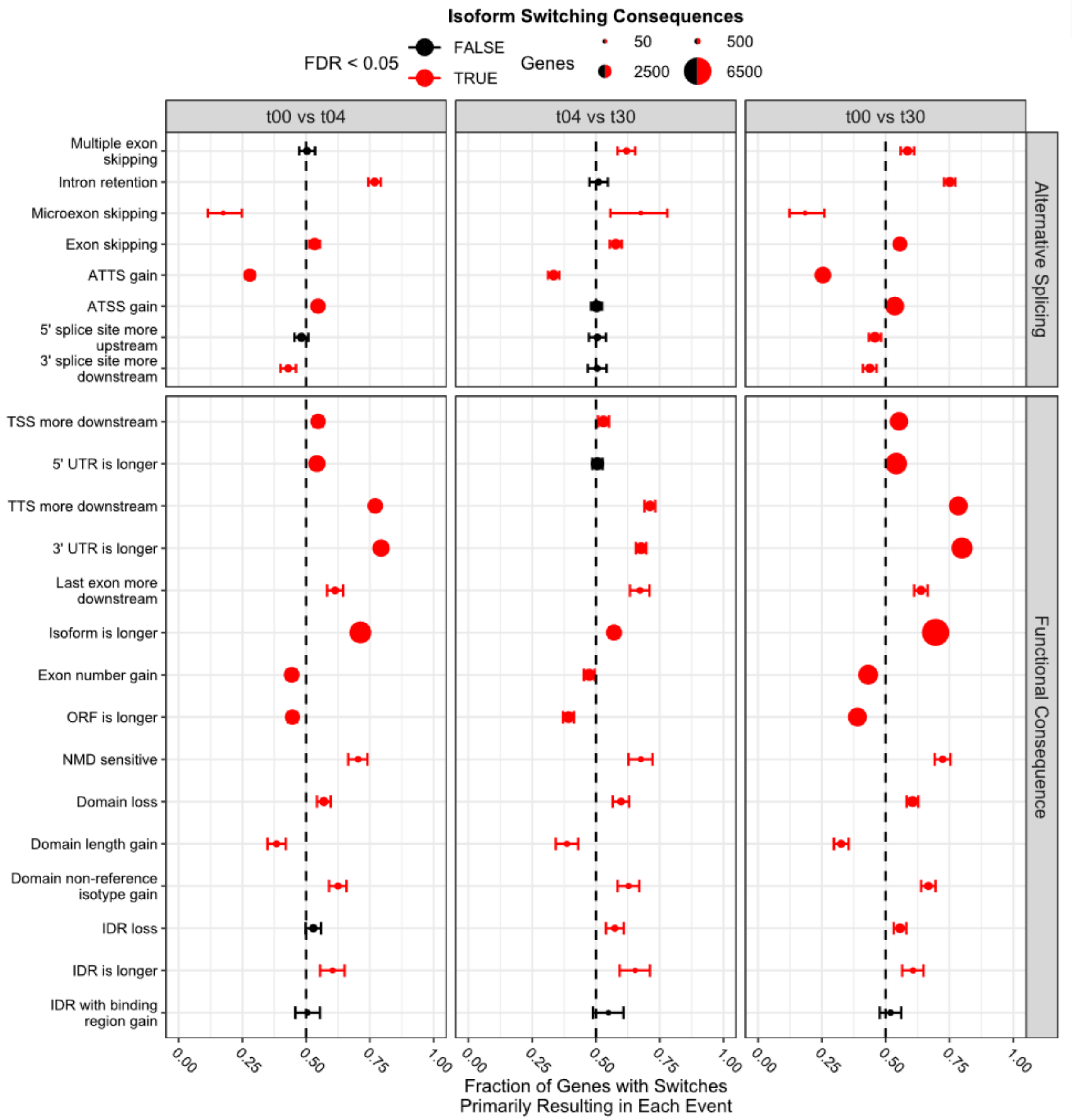

Figure S9. Complete list of isoform switching events for alternative splicing and functional consequences analyzed across differentiation. Points represent the fraction of genes where switches primarily result in a given outcome (e.g., exon skipping). The size of the points represents the total number of genes with isoform switches resulting in either opposing consequence. Error bars represent 95% confidence intervals; significance determined by binomial test.

bioRxiv preprint doi: https://doi.org/10.64898/2025.12.16.693263; this version posted December 19, 2025. The copyright holder for this preprint (which was not certified by peer review) is the author/funder, who has granted bioRxiv a license to display the preprint in perpetuity. It is made available under aCC-BY 4.0 International license.

Fig. S10

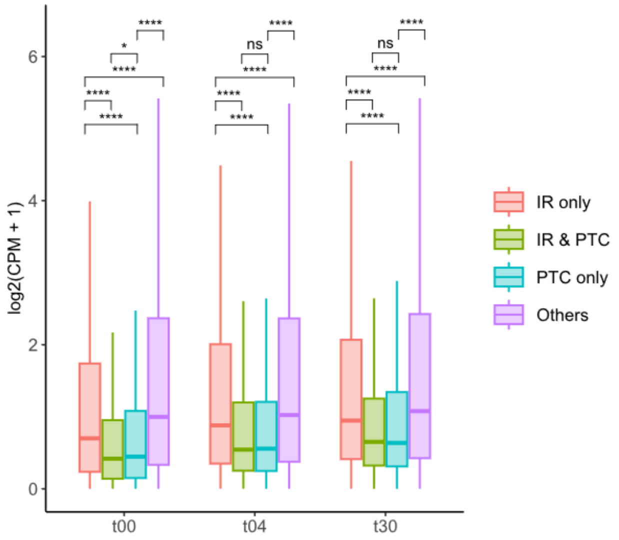

Figure S10. Expression of transcripts grouped by intron retention and premature termination codons. Boxplots compare the expression of transcripts as having Intron Retention (IR) only, IR and a Premature Termination Codon (PTC), a PTC only, and all other transcripts. Statistical comparisons within each time point were performed using a Wilcoxon rank-sum test with Bonferroni correction (*$P < 0.05$; ****$P < 0.0001$; ns = not significant).

bioRxiv preprint doi: https://doi.org/10.64898/2025.12.16.693263; this version posted December 19, 2025. The copyright holder for this preprint (which was not certified by peer review) is the author/funder, who has granted bioRxiv a license to display the preprint in perpetuity. It is made available under aCC-BY 4.0 International license.

Fig. S11

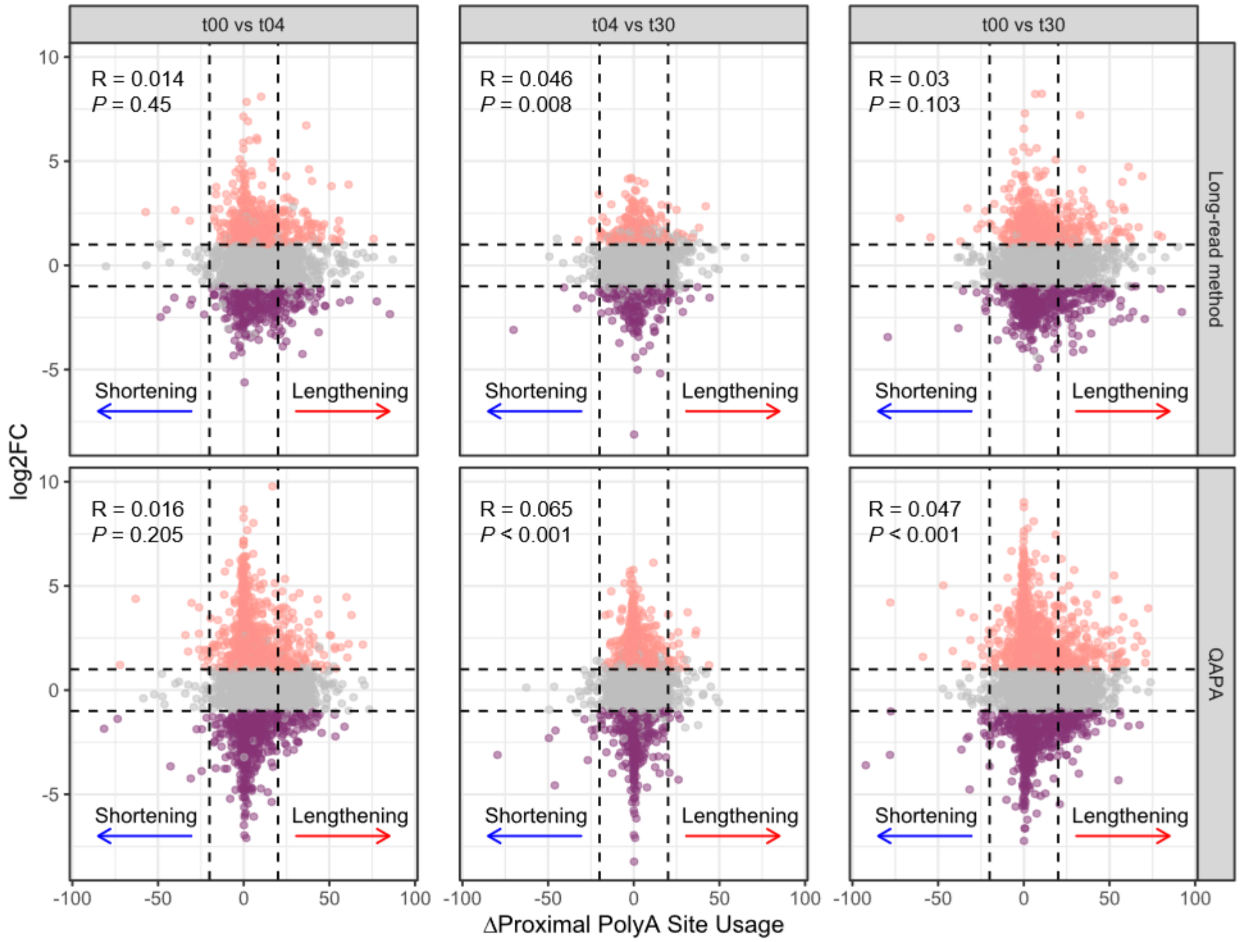

Figure S11. Lack of correlation between 3' UTR length dynamics and mRNA expression changes. Scatter plots compare changes in gene expression ($\log_2$FC, y-axis) against the change in proximal polyA site usage (ΔPPAU, x-axis) between pairs of differentiation timepoints. Top row uses our custom long-read analysis method and bottom row uses QAPA for validation. 3′ UTR shortening (ΔPPAU < −20, blue arrows) and lengthening (ΔPPAU > 20, red arrows) show no clear association with direction of gene expression changes ($|\log_2$FC$| > 1$, FDR < 0.05), including gene upregulation (peach dots) or downregulation (purple dots). Pearson correlation coefficients (R) and *P*-values for all datapoints are shown in each panel. Vertical dotted lines indicate ΔPPAU thresholds, and horizontal dotted lines indicate $\log_2$FC thresholds.

bioRxiv preprint doi: https://doi.org/10.64898/2025.12.16.693263; this version posted December 19, 2025. The copyright holder for this preprint (which was not certified by peer review) is the author/funder, who has granted bioRxiv a license to display the preprint in perpetuity. It is made available under aCC-BY 4.0 International license.

Fig. S12

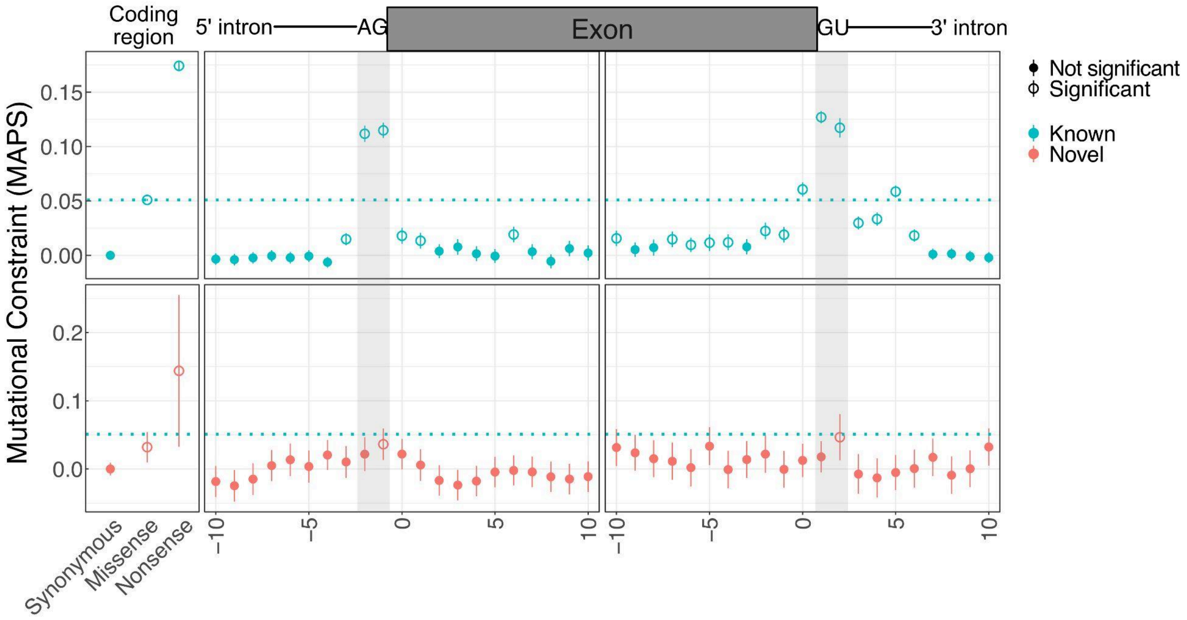

Figure S12. Mutational constraint of coding and near-splice variants using whole-genome sequencing (WGS) data. Related to Figure 6. The plots show mutational constraint (MAPS scores) for single nucleotide variants from 76,215 individuals in the gnomAD v4.1 WGS cohort. The top panel shows constraint at Known (GENCODE V47) elements, and the bottom panel shows constraint at Novel elements identified in this study. For coding consequences (left), scores are shown for synonymous, missense, and nonsense variants. Positions surrounding splice acceptor and donor regions are also shown, with shaded areas highlighting the two essential splice sites. Open circles indicate variant classes or positions with mutational constraint significantly different from synonymous variants (FDR < 0.05, chi-squared test). Dashed horizontal lines indicate the MAPS scores for known missense and nonsense variants, provided for context. Error bars represent 95% CIs.

bioRxiv preprint doi: https://doi.org/10.64898/2025.12.16.693263; this version posted December 19, 2025. The copyright holder for this preprint (which was not certified by peer review) is the author/funder, who has granted bioRxiv a license to display the preprint in perpetuity. It is made available under aCC-BY 4.0 International license.

Fig. S13

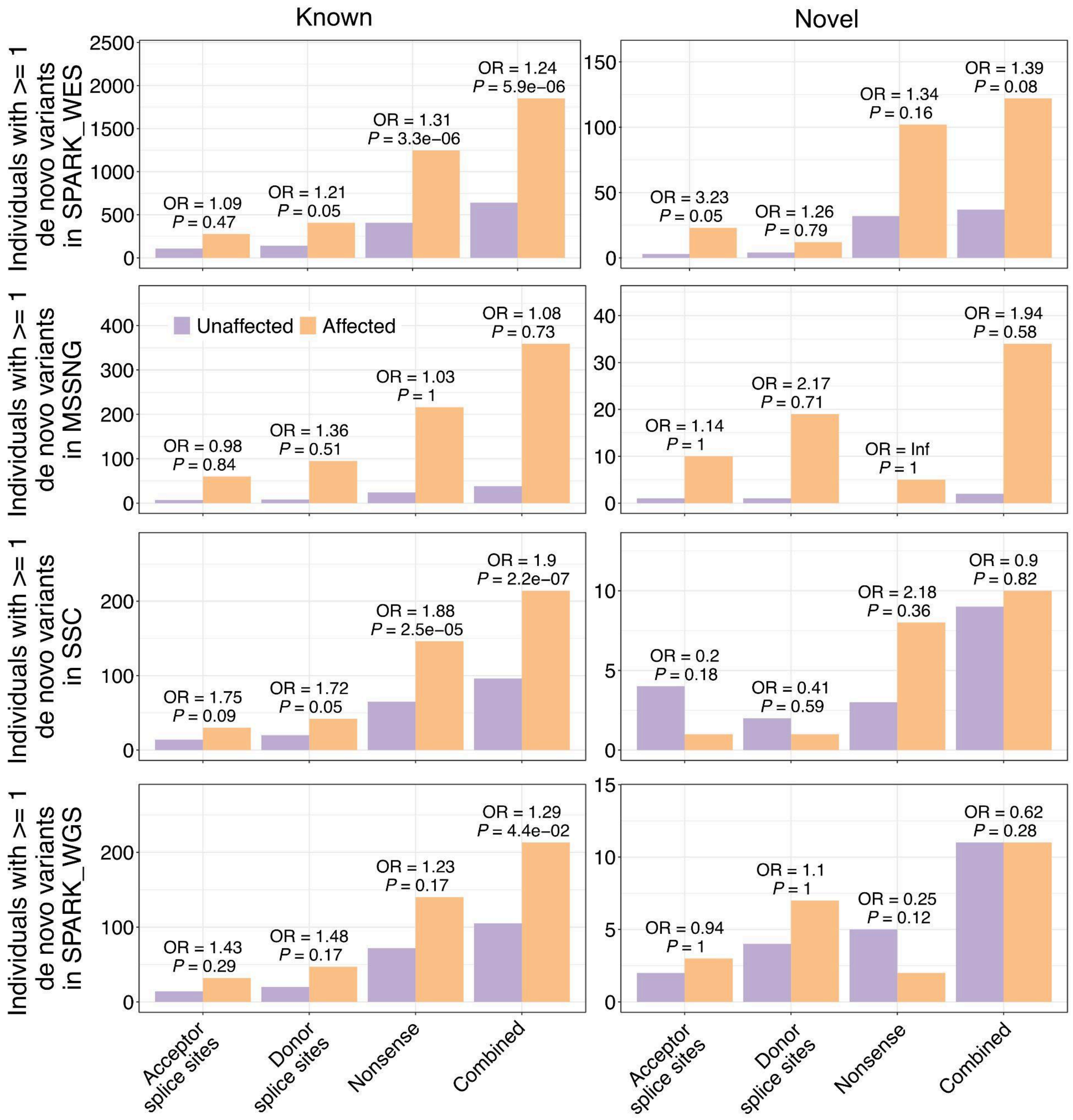

Figure S13. Burden of disruptive *de novo* mutations in individual ASD cohorts. Related to Figure 6. These plots show the burden of *de novo* mutations in individuals affected by versus unaffected by ASD for known (left) and novel (right) genomic elements. Each row represents data from one ASD cohort. Odds ratios (OR) and *P*-values from a Fisher's exact test are shown.

bioRxiv preprint doi: https://doi.org/10.64898/2025.12.16.693263; this version posted December 19, 2025. The copyright holder for this preprint (which was not certified by peer review) is the author/funder, who has granted bioRxiv a license to display the preprint in perpetuity. It is made available under aCC-BY 4.0 International license.

Supplementary Tables

Supplementary Table 1

Supplementary Table 2

Supplementary Table 3

Supplementary Table 4

Supplementary Table 5

Supplementary Table 6

Supplementary Table 7

Table S1. Isoform Atlas and Annotation. Related to Figure 1. This table is derived from classification.txt produced by pigeon classify, which is based on SQANTI3 QC. It is a tab-separated file where isoforms are rows and QC features computed by pigeon classify are columns. For a full glossary of columns and their meaning, see https://github.com/ConesaLab/SQANTI3/wiki/Understanding-the-output-of-SQANTI3-QC#classifcols. It contains 182,371 high-confidence mRNA isoforms identified across the t00, t04, and t30 time points. The table also includes an additional column that indicates the results of the overlap comparison with the external fetal brain dataset [13], where Ensembl or TALON IDs are listed if the isoform identified in our dataset overlaps with one in the Patowary dataset.

Table S2. Catalog of Microexons identified in human iPSC-derived neurons. This table lists the genomic coordinates and classification of 1,930 microexons in 8,008 transcripts (defined as exons < 28 nucleotides) detected in the long-read RNAseq dataset.

- **seqnames, start, end, strand**: Genomic location of the microexon (GRCh38).
- **width**: Length of the microexon in nucleotides.
- **gene_id/transcript_id**: Unique identifier for the PacBio gene ID and transcript isoform containing the microexon.
- **MIC_coord**: Combined genomic coordinates (chr:start-end).
- **GENCODE_v47**: Logical column indicating whether the microexon is found in GENCODE v47 (TRUE), or not (FALSE).
- **vastDB:** Logical column indicating whether the microexon is found in vastDB (TRUE), or not (FALSE).
- **EVENT:** vastDB exon ID.

bioRxiv preprint doi: https://doi.org/10.64898/2025.12.16.693263; this version posted December 19, 2025. The copyright holder for this preprint (which was not certified by peer review) is the author/funder, who has granted bioRxiv a license to display the preprint in perpetuity. It is made available under aCC-BY 4.0 International license.

Table S3. Proteogenomic Validation of Novel Isoforms. Related to Figure 2. This table in .csv file format, lists novel peptides identified by mass spectrometry that provide direct experimental evidence for the translation of protein-coding sequences. It provides the amino acid sequence for each peptide, the corresponding isoform ID(s), and the parent gene.

Table S4. Gene and Protein Expression Dynamics. Related to Figure 3. This table contains the complete gene-level differential expression results and functional enrichment analyses underlying the mRNA-protein dynamics shown in Figure 3.

- **Differential Gene Expression:** Full statistical results from DESeq2 for mRNA-level differential expression between time points (t00, t04, t30).
- **Differential Protein Expression:** Full statistical results from MSstatsTMT for protein-level differential expression.
- **Clusters:** The nine-category expression trajectory assignment (e.g., 'UU', 'D-') for each of the 8,498 genes with both mRNA and protein data.
- **SFARI Gene Enrichment:** Statistical results (odds ratio, *P*-value) for the enrichment of SFARI ASD risk genes in each protein and mRNA cluster, as shown in Figure 3D.
- **GO Term Enrichment:** Complete Gene Ontology (GO) enrichment analysis results for each of the nine protein expression trajectories.

Table S5. Isoform Switching Dynamics and Functional Consequences. Related to Figure 4. This table provides the complete isoform-level differential analysis results and the characterization of their functional consequences, as summarized in Figure 4.

- **DTE (Differential Transcript Expression):** Statistical results (log2FC, FDR) for all isoforms with significant changes in abundance (~49,000 events).
- **DTU (Differential Transcript Usage):** Statistical results (change in isoform fraction 'dIF', FDR) for all isoforms with significant changes in relative usage (~24,000 events).
- **Summarized/Detailed switch conseq/AS:** Gene-level and isoform switch-level characterization of the alternative splicing (e.g., microexon inclusion, intron retention) and functional consequences (e.g., NMD status, protein domain gain/loss) of isoform switching.
- **SFARI Gene Enrichment:** Statistical results (odds ratio, *P*-value) for the enrichment of SFARI ASD risk genes among genes undergoing each class of switching event, as shown in Figure 4C.
- **GO Term Enrichment:** Complete Gene Ontology (GO) enrichment analysis results for genes regulated by specific switching events (e.g., intron retention), as shown in Figure 4D.

bioRxiv preprint doi: https://doi.org/10.64898/2025.12.16.693263; this version posted December 19, 2025. The copyright holder for this preprint (which was not certified by peer review) is the author/funder, who has granted bioRxiv a license to display the preprint in perpetuity. It is made available under aCC-BY 4.0 International license.

Table S6. Coordination of Alternative Splicing (AS) and Alternative Polyadenylation (APA). Related to Figure 5 and Figure S8. This table provides the complete statistical results and supporting data for the analysis of AS-APA coordination and 3'UTR dynamics.

- **Quasi - Results & EMMs:** Contains the full statistical output from the primary quasi-binomial generalized linear model (GLM) approach. This includes the 2,789 significant coordination events, their FDR values for 'global' and 'interaction' effects, and the estimated marginal means (EMMs) values.
- **Fishers - Results:** Contains the complete results from the validation analysis using the contingency table-based Fisher's exact test method, for comparison.
- **QAPA & APA:** Includes the output from the QAPA analysis and the custom long-read APA analysis, detailing proximal polyA site usage (PPAU) and the comparison of dPPAU to gene expression (log2FC) used to generate **Figure S8**.

Table S7. Burden of Disruptive *de novo* Mutations in ASD Cohorts. Related to Figure 6. This table provides QC attributes related to all variants used for the *de novo* variant (DNV) burden analysis, as summarized in Figure 6B. The data is stratified by cohort (MSSNG, SSC, SPARK_WGS, and SPARK_WES) in the “data_cohort” column, and by variant category in the “is_known” column, which is a column that is “True” when the isoform is known, “False” when the isoform is novel.

- **Known:** Variants classified as disruptive using the GENCODE V47 reference.
- **Novel:** Variants reclassified as disruptive *only* by this study's novel transcriptome. This data is used to calculate the odds ratios (OR) and *P*-values (Fisher's exact test) to assess the contribution of novel elements to ASD risk.
